# The effects of a brief intervention at home based on shared reading to promote children’s oral language

**DOI:** 10.1590/2317-1782/e20240003en

**Published:** 2025-01-20

**Authors:** Camila Domeniconi, Marta Gràcia, Priscila Benitez

**Affiliations:** 1 Departamento de Psicologia, Universidade Federal de São Carlos – UFSCAR - São Carlos (SP), Brasil.; 2 Departament de Cognició, Desenvolupament i Psicologia de l’Educació, Universitat de Barcelona – Barcelona, España.; 3 Centro de Matemática, Computação e Cognição, Universidade Federal do ABC – UFABC - Santo André (SP), Brasil.

**Keywords:** Shared Reading, Collaborative Reflection, Mother-Child Interactions, Teaching Strategies, Preschool Children

## Abstract

**Purpose:**

The general aim of the present study was to analyse eight mother-child interactions during shared reading with children and to assess the efficacy of a brief intervention with the mothers to promote changes in the strategies they used to develop their children’s oral language. The specific objectives were to work collaboratively with mothers, to analyse the interactions between mothers and their children before and at the end of the intervention period.

**Methods:**

Mothers participated in five meetings to reflect collaboratively on strategies to promote improvements in communicative interactions in a family context and in children’s oral language and during the shared reading episodes. Standardized language tests were used to assess the oral language of children who were five and six years old and typically developing.

**Results:**

The results showed that all children improved some aspects of their communicative repertoire in standardized tests and/or in the observations of natural or structured situations.

**Conclusion:**

We conclude that the use of strategies such as a brief intervention to promote communicative interactions between mothers and their children may have contributed to promoting children’s oral language.

## INTRODUCTION

Shared reading represents an important mode of interaction between parents (or significant adults) and children, and can promote communication, language development, cognition and emergent literacy skills^(1-3)^. Shared reading activities provide an opportunity to teach new vocabulary, because of the topics and the structure involved in the books, for example poetry books or stories about exotic animals.

During shared reading, adults and children usually succeed in having a relaxed and pleasant conversation that could be beneficial to communicative interaction. While parents are reading a story book and asking their child questions, they also talk with him/her about the story and about the names of the things they can see in the book. These strategies have been related to increased word learning and functional vocabulary.

The strategies employed by parents during shared reading can affect the possibility that the activity will have positive effects on language promotion^(1,4)^. For example, the type of book chosen by the parent is an important variable. Books that are too complex or too simple in terms of structure or content may not have a potential benefit on the child’s language. The same is true for the age of children. It is well known that shared reading can increment the vocabulary of children older than three years. However, few studies have included children under the age of 3^(5,6)^. For this age group, different strategies may be required during the shared reading.

Colmar^(7)^ investigated how a shared reading intervention by parents impacted the vocabulary of children in situations of socioeconomic disadvantage. The participants were 36 children who were divided into experimental and control groups. All children in the experimental group were assessed as having language difficulties. Among the control group, half of the children had been evaluated as typical in terms of language and the others were assessed as having language difficulties. All participants were initially pre-tested for receptive and expressive language skills, using standardized tests. Parents of children in the experimental group were taught how to interact with their children during the daily reading of books, using four components: pauses on each page of the book (to give the child the opportunity to initiate a conversation); open questions for the child about the topic of the book; images as discriminative stimuli to elicit comments from the child; and conversations about the book’s contents in everyday moments, with pauses (it is important to give the child a chance to initiate a conversation). After four months of intervention, all the children were retested. The post-intervention data showed that the children in the experimental group had significant gains in spontaneous language, compared to the pre-test data, which were not observed in any child in the control group.

Domeniconi and Gràcia^(8)^ used the strategy presented by Colmar^(7)^ – during reading and everyday conversations, the parents talk about the book they are reading – as part of an intervention programme focused on families. They assessed the strategy’s effectiveness at improving the oral language of five-year-old children. The four families participated in five meetings with the researcher in which they discussed useful strategies to help children to improve their oral language. The researcher observed the children in different contexts and collaboratively with parents introduced new strategies into their daily routines. The analysis of the data highlights the efficacy of family-focused interventions to promote communication skills.

The strategies used by parents in a natural situation during reading can help researchers, education professionals and speech therapists to structure reading interventions by planning individualized interventions for children with or without disabilities and by working collaboratively with families^(9)^. Questions to consider are the quality of the interaction between mother and child during a shared reading activity at home, the strategies that are used, and whether a brief intervention with families can strengthen and expand children’s language.

The main aim of the present study was to examine mother-child interactions during shared book reading with their children and to assess the efficacy of a brief intervention with the mothers to promote changes in their strategies and in the children’s oral language. The specific objectives were: 1) to detect changes in children’s oral language at the end of the collaborative work with mothers; and 2) to analyse interactions between children and their mothers before and at the end of the intervention period. The study was designed to discuss how to reflect collaboratively with mothers.

A brief intervention with the counseling family to reflect on opportunities for language development in their homes with their children proved useful for this group of participants. The present study extends the scientific contributions of family-centered interventions and advances in relation to systematization in a brief counseling format, in a partnership between researcher and family.

## METHOD

### Participants

The participants were eight children (three girls and five boys), aged 5–6 years (last level of kindergarten) and their mothers. All of them came from a low-middle class economic background and attended a state school located in a city of 150,000 inhabitants in the province of Barcelona. The project was presented at the school that the children attended and families who were interested and available participated. The intervention was proposed for this age group because it is an age in which the transition from preschool to elementary school, with changes in demands and routines, becomes a concern and a reason for reflection for parents.

The characteristics of the participants’ parents are presented in Table 1. As shown in this table, all parents involved in the intervention had a secondary or technical educational level, which guaranteed full understanding of the proposed strategies and discussions. The family had children’s books at home but had poor reading and shared reading habits.

**Table 1:** Characteristics of the participants

Name(s) of child	Alan and Ian (twins)	Paulo	Gustavo	Lidia	Livia	Pedro	Lara
**Child’s age (years)**	5	5.6	5.9	5.11	5.7	6.0	5.9
**Child’s gender**	Male	Male	Male	Female	Female	Male	Female
**Family status**	Both parents	Both parents	Both parents	Both parents	Both parents	Single mother	Both parents
**Parents’ age (years)**	38, 38	40, 44	25, 29	38, 41	37, 39	46, 48	27, 33
**Mother’s education**	Professional training	Professional training	Secondary	Professional training	Professional training	Secondary	Professional training
**Father’s education**	Secondary	Secondary	Secondary	Postsecondary	Secondary	Postsecondary	Postsecondary
**Shared reading**	No	Yes, every night	No	No	Sometimes	No	Sometimes
**Books at home**	Yes	Yes	Yes	Yes	Yes	Yes	Yes
**Parents’ reading activity**	Newspaper (father)	No	No	No	Sometimes	No	No

The study was submitted and accepted by the Ethics Committee of the University Federal of São Carlos (CAAE 55340016.0.0000.5504). Families were informed about the study procedures and provided informed consent.

### Instruments

- Oral language assessment instruments

To analyse the children’s communicative and linguistic abilities, the following instruments/approaches were used.

1. The Navarra Oral Language Test (PLON)^(10)^ and the pragmatic part of the Revised BLOC Screening Test (BLOC-SR)^(11)^. The two instruments were applied in accordance with the standards of each instrument and were audio-recorded for later review, as required.
2. An instrument to assess the children’s conversational skills. The instrument used to assess the conversational skills of children in their natural environment was developed within the framework of the project, funded by EVALOF and conducted by the author and colleagues. This instrument allows the assessment of non-oral (4 items) and oral abilities (18 items grouped into 6 areas of communicative ability: shift management, coherence, communicative functions, argumentative discourse, formal aspects and use of the school’s vehicular language) of each child in a conversation situation. The conversation occurred in groups of 3–5 children who were invited by the researcher to draw and paint in a comfortable space at the school.
3. Analysis of a book-reading episode (child-researcher). The situation always followed the same structure: 1) the child was invited to read a story with the researcher in a school space; 2) the researcher offered three book options (all three very similar in terms of type, number of pages and the quantity of text on each page) and the child had to choose one; 3) the researcher read aloud the title of the book, opened the book to the first page and waited for the child to start saying something and turn the pages;
4. After finishing the book, the researcher always asked the child to summarize the story.

The categories that were assessed by analysing the video recordings of this activity were: 1. Handling (manipulation): evaluates whether the child handles the book, looks at the images and/or opens pages; 2. Saying something during pauses (pauses): measures whether the child says something about each page of the book; 3. Connectivity (connective): assesses the child’s skills at telling a story in a certain sequence that follows the pages, including the use of connectors (for example, then, so and after); 4. Conclusion (finish): evaluates whether the child finishes the story with some logic (in relation to the story) or the use of word “end”; 5. Synthesis (synthesis): indicates the ability to incorporate the summarizing skill when requested by the researcher; 6. Narration (narration): the stories a child told were analysed and grouped according to the parameters of narrative skills, as expected for the children’s age. The strategies could have repercussions on qualitative aspects of children’s performance during shared teaching. Categories 2, 4, 5 and 6 were taught directly, while 1 and 3 are conditions that assess the quality of the child’s narrative production when they handle books. These conditions were not explicitly taught but were used during the activity.

To assess the routines and communicative interactions of mothers with their children, the following instruments/approaches were used:

1. Before the intervention, a script was used to carry out an interview with families about their daily routines based on McWilliam’s^(12)^ proposal.
2. EVALOF^(13)^. The Scale of Oral Language in Familiar Context Assessment (EVALOF) is a tool adapted from the Scale of Oral Language in School Context Assessment (EVALOE)^(14)^. It consists of 32 items grouped into two subscales: 1) Context and better management of communication (12 items); and 2) Communication functions and strategies (20 items). Each of the items can be assessed by the observer with a score of 0 to 4: 0 – cannot be observed (this option must be chosen if, for some reason, the item cannot be observed, for example, if the question refers to a network interaction between a small group of people, and only two people were present, the item cannot be observed); 1 – the item is not observed; 2 – the item is sometimes observed; 3 – the item is observed systematically or almost always. This version of the EVALOF was completed in the present study by viewing video recordings of episodes of shared reading with a child and their mother. Families were asked to videotape an episode that was as natural as possible. Observations were carried out before and after the intervention.

### Intervention programme

Five sessions were planned with all mothers. The video recordings (undertaken by the researcher) and meetings were carried out in the home, and the rest of the phases of the collaborative procedure were completed at school. The duration of each interview was between 1 hour (individual) and 1 hour 30 minutes (group), with a bi-weekly frequency. The topics for each session were planned according to a sequence of strategies to be discussed collaboratively with the mothers, to promote improvements in the children’s oral language and in the communication interactions in the family context.

The intervention was based on Domeniconi & Gràcia^(8)^. The sequence of discussion of each strategy is presented in Table 2. Mothers were encouraged to use the strategies discussed in a book-reading context for 5–15 minutes every day with their child.

**Table 2:** Strategies implemented during the intervention, description and session

Strategies	Description	Session
1. Allowing pauses on each page	This strategy gives the child the opportunity to talk, with the support of a figure in the book. This strategy also allows the adult to follow the interest of the child, since it starts the story on each page.	1
2. Asking open questions related to the child’s chosen topic	Open questions allow for broader conversations, better-structured phrases and the use of a variety of word classes.	1
3. Following the child’s interest while they look at the books and at other times of conversation	By pausing on each page, the adult allows the child to lead the story and can follow their interest, maintain motivation and stimulate imagination and creativity.	2
4.Alternating turn- taking in conversation	This provides the child with the time needed to speak and strengthens the importance of changing shifts, a skill that they will need in all other conversations.	2
5. Adapting the language	With appropriate adaptations in language, the adult can maintain the child’s interest in the story and ensure the child’s understanding of the plot, even when they are very small. These are adaptations in words, looking for more accessible synonyms that are part of the child’s universe, and adaptations in the structures of sentences, which are shorter and more direct.	3
6. Expanding and clarifying	This strategy teaches adults how to take advantage of what the child says and, from there, expand what they say and clarify if necessary. The strategy aims to maintain the conversation based on what is said by the child but also to expand his/her communication repertoire and provide a model, in case of incorrect utterances, without the need to correct it. For example, if a child looks at the picture in the book and says “Oia a buóia” the adult can say: “Yes, look at the red ball, isn’t it beautiful?”	3
7. Teaching how to summarize	Summarizing and drawing conclusions is an important communication skill. Through this strategy, the adult teaches the child to pay attention to the essential aspects of a narrative and the essential elements for understanding the story. It favours the formulation of conclusions, opinions and critical thinking.	4
8. Teaching how to self-evaluate	The goal is to encourage children to evaluate their own communication repertoire. This is achieved through questions such as: “Do you think that if you tell the story to Grandma she can understand it?” The aim is for the child to evaluate his/her performance and thus improve his/her communication repertoire.	4

In the first session (individual meetings), the researcher discussed with each mother some of the details of natural communication interactions that already took place at home. This was based on an analysis of the routine video observations and the Family Routine Interview^(12)^, in particular regarding the conditions and the motivation to read by the child and the mother. After the first session, each session (sessions 2 to 4) followed the same structure: 1) Discussion about the use of the strategies suggested in the previous session (frequency of reading, difficulties in the use of strategies, children’s engagement); 2) Presentation of new strategies, suggesting their use in the coming days. The presentation could involve written definitions (for example, a definition of expansion and clarification), audio and video examples or other strategies; 3) Agreement about the timing of the next session.

### Data analyses

The linguistic competence of the children was assessed using the Navarra Oral Language Test (PLON), the pragmatic part of the BLOC test, observation of conversations between children, observation of conversations between each of the children and a family member, and observation of a reading situation with the researcher.

The index of inter-rater agreement (independent first and second raters) was calculated to score the strategies used by mothers and the responses given by children during shared reading. The video recordings used to calculate agreement were the interactions between Iuri, Alan, Paul and Noah and their mothers, in the post-tests. To perform the calculation, the number of agreements was divided by the number of agreements plus disagreements, multiplied by 100^(15)^. The result was a general agreement of 95.2%.

## RESULTS

**Figure 1:**
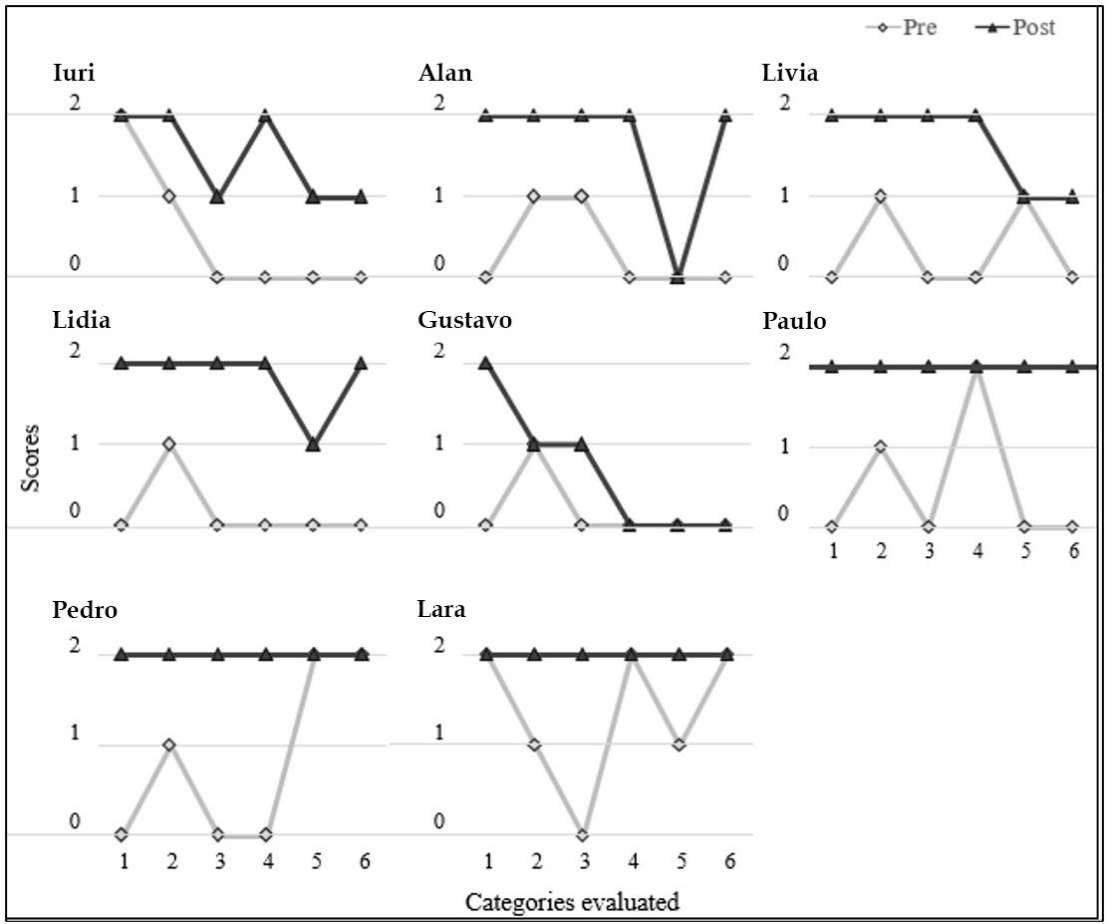
Pre and post-test performance of children in the categories during shared reading with the researcher

First, we present the results related to the observations made during book- reading episodes with the researcher. Each skill was analysed according to frequency of emission: 0 - the skill was not observed; 1 - the skill was sometimes observed; 2 - the skill was systematically observed. Figure 1 shows the number of participants that used each analysed skill with relatively high frequency (that is, grade 2 or higher).

**Caption:** Categories - 1: manipulation, 2: pauses, 3: connective, 4: finish, 5: synthesis, 6: narration

Figure 1 shows that more children engaged in actions/activities that could be considered interesting and important in a shared reading situation after the intervention. The categories of synthesizing (after reading) and developing a kind of narrative (while reading) were observed in four out of five children in the post-intervention.

Tables 3 and 4 present the most important outcome linked with the main objective, because it is the outcome that is most directly related to the intervention: mothers discussed collaboratively with the researcher some strategies to use in interactions when they read stories to their child. Mothers were then observed to detect whether they used these strategies and whether the child also learnt them.

**Table 3:** Results obtained using the EVALOF tool to observe shared reading interactions before and after the intervention

	Pre	Post	Agreement post-test	Participation in intervention sessions (Maximum score=5)
Iuri and Alan	63 (49%)	85 (66.6%)	88.7%	5
Gustavo	55 (42.9%)	64 (50%)	-	3
Livia	72 (56.25%)	81 (63.2%)	-	5
Lidia	71 (55.4%)	86 (67.1%)	98.7%	5
Paulo	63 (49%)	75 (58.5%)	95.2%	5
Pedro	75 (58.5%)	82 (64%)	-	5
Lara	68 (53.1%)	75 (58.5%)	-	3

**Table 4:** Strategies that were more and less used by mothers during shared reading in the post-test, with the application the EVALOF

Strategy	Mother’s behavior	Child’s behavior	Score/ Classification
*Subscale: 1. Communication context and management*			
During the activity, the adult positions herself and organizes the environment to adapt to the characteristics of the activity performed by the child	✔		18 / Most
During the activity, the more refined physical context (lighting, noise, connected devices, use of mobile phones or computers, etc.) facilitates communicative interaction	✔		18 / Most
The adult gives the child time to participate in the communicative interaction	✔		18 / Most
The adult responds to communicative interactions initiated by the child	✔		18 / Most
The child finds it easy to get the adult to focus on his/her communicative initiations		✔	18 / Most
The child manages his/her participation in the conversation spontaneously, without any adult asking him/her to participate		✔	17 / Most
The adult helps the child to initiate communicative interactions	✔		17 / Most
The adult is responsive to communicative interactions initiated by the child	✔		16 / Most
The child is responsive to communicative interactions initiated by the adult		✔	16 / Most
*Subscale: 2. Communicative functions and strategies*			
The child requests information		✔	18 / Most
The adult gives attention and/or provides the information requested by the child	✔		18 / Most
The adult takes advantage of the ongoing activity to work on aspects of oral language with the child	✔		17 / Most
The child provides information		✔	16 / Most
The child uses norms of social interaction		✔	16 / Most
The adult expands the child’s statements	✔		16 / Most
The adult specifies the necessary prior knowledge linked to the current interaction	✔		16 / Most
The child improves his/her utterances from the expansion by the adult		✔	15 / Most
The adult clarifies the content that the child did not understand in the interaction	✔		15 / Most
The adult positively evaluates the content presented by the child	✔		15 / Most
The adult requests the child’s self-assessment	✔		06 / Less
The child does not self-assess their behaviour		✔	06 / Less

Note: Most used = from 15 to 18 points, Medium used = from 14 to 9 points, Least used = from 8 to 6 points

As shown in Table 3, all mothers used more strategies to teach oral language after the intervention, according to the EVALOF items. The mothers achieved the highest scores in the post-intervention test on items such as teaching their children to ask for information, teaching them to synthesize and asking for self-assessment. For some mothers (those of Livia, Iuri and Adam), the strategies of providing clarification and expanding on topics of interest were observed more frequently in the post-intervention test. Paulo’s mother also engaged more with strategies to take turns in conversation after the intervention.

Regarding participation in the intervention sessions, five out of seven mothers attended all sessions. As shown in Table 3, two of the seven mothers were absent from two meetings for personal reasons (doctor’s appointment or family illness). The strategies that were addressed in the sessions they missed were delivered on paper at the next opportunity to meet, and the researcher made herself available for questions.

Table 4 shows the strategies that were most frequently used by mothers during shared reading and the least used in the post-test assessment with three mother-child dyads using EVALOF. In Subscale 1, all strategies were used and in Subscale 2, all strategies but one (self-assessment) were used. The score was based on the sum of the two raters’ scores (minimum = 6 and maximum = 18 for each item). Table 4 presents the results obtained using the EVALOF tool, before and after the intervention.

Second, we present the results obtained in standardized language tests and conversations between the children and their mothers before and after the intervention.

Table 5 presents the results obtained using PLON, BLOC (pragmatic) and group conversation among children (analysed using an *ad hoc* instrument).

**Table 5:** Results obtained by each child participant in PLON and BLOC tests and conversations before and after the intervention

Test	Iuri	Alan	Livia	Lidia	Gustavo	Paulo	Pedro	Lara
PLON* (N = 13)								
Pre	NI (2)	NI (2)	NI (2)	D (3)	D (3)	N (1)	N (1)	N (1)
Post	N (1)	N (1)	NI (2)	N (1)	NI (2)	N (1)	N (1)	N (1)
BLOC (N = 23) Pre	10	8	7	14	8	15	10	10
Post	18	22	19	20	18	21	22	21
Conversation (57 items in total)								
Pre	22	26	19	9	23	21	31	17
Post	41	57	55	39	38	44	56	54

* D: delayed

NI: needs improvement; N: normal

As shown in Table 5, the scores obtained in the post-interventions indicated an increase in language skills in comparison to the pre-intervention scores. Iuri, Alan, Lidia and Gustavo showed improvements in all measures. Livia, Paulo, Pedro and Lara showed improvements in two of the three measures (BLOC and conversation). Notably, Paulo, Pedro and Lara obtained the highest possible scores in the PLON test in the pre-intervention and this result was maintained in the post-intervention assessment.

## DISCUSSION

The main aim of the study was to examine mother-child interactions during storybook reading with preschool children and to assess the efficacy of a brief intervention with the mothers to promote the children’s oral language skills.

The changes related to oral language skills were measured using two standardized tests (PLON and BLOC) and analysing children’s skills under three conditions: shared reading with their mothers, group conversation among children, and shared reading with the researcher. Each pre-intervention and post-intervention situation was recorded and some were later reviewed to check the accuracy of the scoring.

When the children’s performance was compared in the pre- and post-tests, all children obtained higher scores in the post-test in the measures obtained by applying the BLOC test and conversation analysis. In a general analysis of the categories measured during the reading episodes, all children had started to handle the book in the post-test, and seven used the categories *pauses*, *connectors* and *finish* (Figure 1). All participants performed better in one or more measures when the post-test was compared to the pre-test. The category with the highest score (2) in all cases in the post-test was *manipulation.* With the exception of Gustavo, all children had maximum scores for *pauses*. Paulo, Pedro and Lara had maximum scores in all categories in the post-test (see Figure 1).

In EVALOF, all dyads had higher scores in the post-test than in the pre-test assessment. Based on these data, it can be hypothesized that the mothers used all the strategies at some point during the intervention, in response to the performance of all children in the four assessments mentioned. The strategies that were used most by mothers according to the EVALOF application (Table 5) were: 1) organization of the environment, 2) pauses, 3) responding to the child’s communicative interaction, 4) facilitating the initiation of communicative interactions and responding to them, 5) providing information, 6) attention to aspects of oral language, 7) expanding the children’s statements, 8) specifying prior knowledge, 9) explaining the knowledge and 10) positively evaluating the content. Depending on the use of the strategies adopted by the mother, the children: 1) called their mothers’ attention to the communicative initiation, 2) spontaneously managed their participation, 3) reacted to the mothers’ communicative interactions, 4) provided the information requested by the mothers, 5) used norms of social interaction, and 6) expanded knowledge.

In general, the use of the strategies included in EVALOF increased during the period of intervention. This means that strategies such as allowing pauses on each page, following the child’s interest while they look at the books or alternating turn-taking in conversation were useful, because they are related to some strategies included in Subscale 1 (the adult gives the child time to participate in the communicative interaction, the adult responds to communicative interactions initiated by the child, or the child finds it easy to focus the adult’s attention on his/her communicative initiations). However, strategies such as teaching how to summarize or teaching how to self-evaluate were less useful because they were introduced at the end of the intervention period, and because they were more complex for the children and probably far from a usual style of conversation. Some studies reported that these strategies are also difficult to detect in the school context^(16)^.

These results are consistent with those of previous studies^(3,4,8,17)^ involving parents and educators. They confirm the importance of encouraging parents to engage in regular shared reading and to improve their knowledge on how to promote language development through shared reading by considering strategies that can improve the educational potential of this activity.

Zuanetti et al.^(3)^ also described the results of an intervention based on a shared reading programme (comprised of 15 meetings) and compared the results between a comparison group (27 children) and an intervention group (17 children). Statistical analysis was applied and showed that children in the intervention group showed significant improvement in the evaluated variables. Other studies used control group designs to verify the relationship between the intervention and the results obtained in shared reading programmes^(3,7,18,19)^.

Our results coincide with the outcomes reported in a study by Brown et al.^(17)^ on the perception of 113 parents of babies and toddlers about shared-reading activities, specifically the strategies they used to choose a target book and the strategies they used when reading with their children. The authors found that the parents usually engaged in shared-reading activities with their children, but reported difficulties in book selection and strategies for facilitating babies’ early communication development during shared reading.

For example, the participants described the positions usually adopted during reading, with most of them (46%) placing the child on the parent’s lap, with their back to the parent, facing the book. A simple change in this reading environment could produce better language promotion results, given the importance of eye contact and joint attention for building early communication skills.

The only strategy that was not used by any of the mothers was requesting the child’s self-assessment after shared reading (Table 5). We recommend that future studies should create more specific conditions for implementing this strategy during shared reading. Specifically, strategies should be introduced to increment reflection about language and about how children and adults use it in the family and school context. Reflection about self-abilities on language are useful competences to continue progressing in oral and written language development in any context.

Our interview on routines showed that three out of seven mothers usually engaged in shared-reading activities before the intervention (Table 1 shows that Pau did share reading every night, Nerea and Lucia sometimes did, and the others did not; we did not have any information about school activities). This was not consistent with the results reported by Brown et al.^(17)^, who found higher levels of engagement in shared reading. There are many differences between the study by Brown et al.^(17)^ and ours, such as the children’s ages, the country of study, the number of participants, the measurement tools (interview vs. questionnaire) and sociocultural aspects of the parents. Probably one of the main differences between both studies is that the families in our study came from a low-middle class economic background and none of the mothers had post-secondary studies.

Domeniconi and Gràcia^(8)^ showed the possibility of performing brief interventions with low-income family members and obtained interesting results, especially in the promotion of communicative skills in children. In their study, the topics and themes of discussion were proposed mainly by the parents in individual meetings, and the overlap between themes and the strategies used by them was used to plan a group intervention.

These findings are consistent with those of previous studies that examined home-based reading between mothers and children with or without disabilities^(18,20)^. Further studies could assess the skills reported by the previous study^(21)^ to present positive relations after shared reading, such as imagination, social interactions, affect, prosocial behaviour and social play. These studies could apply the intervention either at school or in the family home, to assess whether this difference contributed to the effects on children’s oral language development.

The present study shows that a brief five-session intervention with mothers was able to improve the oral language performance of the participating children, in a naturalistic setting, as shown by previous studies on a similar issue and with a similar population^(8,22)^. The involvement of mothers in the intervention replicates previous studies, and the structure of the intervention and the care taken with the assessments of children and mothers guarantee the accuracy of the findings. It is important to try to understand the potential and limits of the effectiveness of brief interventions, which save time and human resources. Brief interventions can be more easily generalized for use by schools and in public environments to support families by sharing simple, effective strategies that are scientifically tested and can have an impact on promoting children’s language.

Finally, the reading strategies used by mothers in this study could be further analysed in future studies, considering, for example, individualized strategies according to the age of the children (with older children, more “dialogic” strategies could be effective)^(5)^ and improving the use of the “pause” strategy. Read et al.^(23)^ described better results of the pause strategy to learn new words when the pause is used to predict the word than if the pause is used to repeat the new word. These are examples of possibilities to improve the advantages of shared reading strategies and they can be assessed.

Another important naturalistic setting of children is the school. Menotti et al.^(16)^ showed the results of the use of five shared reading strategies by pre-school teachers to promote the quality of teaching oral language in school, considering the shared reading episodes. The teachers were randomly distributed in an intervention group and a control group, and the results obtained from application of the Assessment Scale of Oral Language Teaching in School (EVALOE) indicated positive modifications of teachers’ strategies during shared reading episodes.

## CONCLUSION

Despite the positive results of the present study, they do not allow a definitive conclusion about the role of the intervention in improving the linguistic repertoire of

children. This is because the research design did not enable an analysis of the intervention’s impact separately from the effects of the children’s participation in other activities and interactions with older children, teachers, extended family and others. The positive results of the study should also be considered with caution because the children were at an age of typical rapid oral language development. Although the aim of the study was to carry out an individualized intervention^(9)^, future studies could use a group design with control and experimental groups^(3,7,18,19)^.

Future studies could obtain measurements of other important aspects of emotional conditions of parents and family interactions that are probably affected by an intervention such as that in present study. Therefore, considering the main aim of the study, which was to examine parent-child interactions during book reading, the results show the positive effect of these interactions on oral language development in children.
